# Acceleration of Longitudinal Track and Field Performance Declines in Athletes Who Still Compete at the Age of 100 Years

**DOI:** 10.3389/fphys.2021.730995

**Published:** 2021-09-28

**Authors:** Bergita Ganse, Anne Kristin Braczynski, Christoph Hoog Antink, Matthias Knobe, Tim Pohlemann, Hans Degens

**Affiliations:** ^1^Division of Surgery, Werner Siemens Foundation Endowed Chair of Innovative Implant Development, Saarland University, Homburg, Germany; ^2^Department of Trauma, Hand and Reconstructive Surgery, Saarland University, Homburg, Germany; ^3^Department of Neurology, RWTH Aachen University Hospital, Aachen, Germany; ^4^Institut für Physikalische Biologie, Heinrich-Heine University Düsseldorf, Düsseldorf, Germany; ^5^KIS*MED - AI Systems in Medicine, Electrical Engineering and Information Technology, TU Darmstadt, Darmstadt, Germany; ^6^Department of Orthopaedic and Trauma Surgery, Lucerne Cantonal Hospital, Lucerne, Switzerland; ^7^Research Centre for Musculoskeletal Science & Sports Medicine, Manchester Metropolitan University, Manchester, United Kingdom; ^8^Institute of Sport Science and Innovations, Lithuanian Sports University, Kaunas, Lithuania

**Keywords:** aging, master athletics, physical activity, longevity, oldest-old, centenarian, javelin throw, long jump

## Abstract

While physical performance decline rates accelerate after around the age of 70 years, longitudinal athletic performance trends in athletes older than 95 years are unknown. We hypothesized a further accelerated decline in human performance in athletes who still perform at the age of 100 years. To investigate this, longitudinal data of all athletes with results at or over the age of 100 years were collected from the “World Master Rankings” data base spanning 2006–2019 (138 results from 42 athletes; 5 women, 37 men; maximum 105 years) and compared to previously published longitudinal data from 80- to 96-year-old athletes from Sweden (1,134 results from 374 athletes). Regression statistics were used to compare performance decline rates between disciplines and age groups. On average, the individual decline rate of the centenarian group was 2.53 times as steep (100 m: 8.22x; long jump: 0.82x; shot put: 1.61x; discus throw: 1.04x; javelin throw: 0.98x) as that seen in non-centenarians. The steepest increase in decline was found in the 100-m sprint (*t*-test: *p* < 0.05, no sign. difference in the other disciplines). The pooled regression statistics of the centenarians are: 100 m: *R* = 0.57, *p* = 0.004; long jump: *R* = 0.90, *p* < 0.001; shot put: *R* = 0.65, *p* < 0.001; discus throw: *R* = 0.73, *p* < 0.001; javelin throw: *R* = 0.68, *p* < 0.001. This first longitudinal dataset of performance decline rates of athletes who still compete at 100 years and older in five athletics disciplines shows that there is no performance plateau after the age of 90, but rather a further acceleration of the performance decline.

## Introduction

Human longevity, limits of the human life span and physical performance in old age are of great interest due to the increasing proportion of older people in western societies. It has been speculated that the human mortality rate reaches a “plateau” after the age of 105 (Barbi et al., 2018) but it is unknown whether a plateau, or attenuated decline, also exists for physical performance. Frailty that is associated with mobility limitations and loss of autonomy is a major cause of a poor quality of life of the oldest old (Portegijs et al., 2016; Ding et al., 2017). Indeed, for the oldest old, key to a good quality of life is physical independence (Arai et al., 2014). Data of the few extraordinary individuals who are still able to compete in sports at an age where most people are unable to move and care for themselves are therefore of particular interest for the older people themselves, healthcare providers and insurers, as they may not only inspire other older people, but also show the limits of the physiologically achievable in the oldest old.

In the master athlete population, performance decline rates based on results from annual rankings are currently known up until the age of 95, but not beyond (Ganse et al., 2020a). Analyses of such databases have revealed that the rate of decline of physical performance is initially almost linear, and then accelerates from around the age of 70 years (Da Silva Aguiar et al., 2020). This acceleration has not only been shown in cross-sectional (Young and Starkes, 2005; Ganse et al., 2018), but also in longitudinal data (Lazarus and Harridge, 2017; Ganse et al., 2020b). The decline rate accelerates even further beyond the age of 80 (Ganse et al., 2020b). However, while in longitudinal data most individuals show an acceleration, some athletes have a slower or faster performance decline than others (Donato et al., 2003; Rubin et al., 2013; Ganse et al., 2020b; Hoog Antink et al., 2021). Only few cross-sectional data of centenarian athletes are available (Lepers et al., 2016), but there are no longitudinal data on athletes around 100 years, despite the need for more knowledge in the field of frailty and aging-research (Arai et al., 2014; Portegijs et al., 2016; Ding et al., 2017). Increased participation of centenarians in competitive sports now allows us to assess the rate of performance decline also in a population of oldest-old athletes.

Based on the observation that world records up to the age of 105 years suggest a progressive age-related acceleration of performance decline in many disciplines (Baker and Tang, 2010), we hypothesized a further acceleration of the decline rate in performance of master athletes that are around 100 years of age. To determine whether the rate of performance decline in master athletes is accelerated after the age of 90 years, we combined the data from 80+-year-old athletes in 5 disciplines from a longitudinal data set we published recently (Ganse et al., 2020b) with new longitudinal data of athletes who still competed at age 100 years or older from the database “World Master Rankings.” This represents the first master athletics performance data set ever published of this age group.

## Materials and Methods

Ethical approval was obtained from the IRB of Saarland Medical Board (Ärztekammer des Saarlandes, application number 135/21).

### Generation of Data Set

Performance data of all athletes with results at age 100 years and older were collected from the publicly available “World Master Rankings” data base (www.mastersrankings.com). As the data base reached back to the year 2006, and since restrictions due to the COVID-19 pandemic led to the absence of data in the year 2020, results of both sexes from 2006 to 2019 were considered. For all athletes who had a result at an age of 100 years and older, the data base was searched for additional results from previous ages going back to as early as the age of 90 years to obtain a longitudinal trajectory of the individual.

### The Swedish Data Set

The new data were compared to the data of all 80+-year-old athletes in a data set from Sweden that was published previously (Ganse et al., 2020b). Briefly, this data set comprises all data from the Swedish master athletics rankings from the years 1901 to 2019 and is the largest longitudinal master athletics data set published to date.

### Implements

Changes in weights of javelins, discusses, and shots with age did not affect the results in the present analysis, as implement weights in the throwing disciplines stay constant for athletes 80 years and older. The following implements are used by athletes 80 years and older: shot put: men 3 kg, women 2 kg; discus throw: men 1 kg, women 750 g; javelin throw: men and women 400 g.

### Statistical Analysis

All statistical tests were executed with IBM SPSS Statistics version 25. Regression statistics were employed to compare performance decline rates between disciplines and age groups. Regression coefficients and their corresponding *p*-values were calculated (significance level 0.05). Disciplines were included in the study if the following inclusion criteria were met: at least three athletes with at least two results in the data set and at least one result at an age of 100 years or more. Linear regression decline rates were computed for the younger (the data from Sweden) and the older (the data of the athletes who still compete at 100 years) data set separately to allow direct comparison of declines, even though other regression types would have delivered higher *R*^2^-values. Two separate analyses were conducted: linear regression on all pooled data points of each group, and linear regression on the individual regression lines of each athlete, combined with *t*-tests. We chose linear regression, as the optimal type of regression function differed between disciplines, and to be able to compare decline slopes between the centenarians and the non-centenarian old athletes.

## Results

### Characterization of the Data Sets

We collected 138 results from 42 athletes (5 women and 37 men) of whom results at the age of 100 years or more up to the age of 105 were present in the data base (Table 1). The most data in this age group were available from discus throw and shot put, followed by javelin throw, 100-m sprint and long jump. Table 1 also shows the numbers of athletes and results in the disciplines in the other longitudinal data set of athletes between 80 and 96 years of age with 1,134 results from 374 athletes.

**Table 1:** Numbers of athletes and data points per discipline in the present centenarian (100+-years group) and Swedish (80+ group) data sets, separated by sex.

	**Number of centenarian athletes**			**Number of centenarian data points**		
**Discipline**	**Men**	**Women**	**Total**	**Men**	**Women**	**Total**
100 m	7	3	10	18	6	24
Long jump	3	0	3	10	0	10
Shot put	12	1	13	36	2	38
Discus throw	9	0	9	39	0	39
Javelin throw	6	1	7	23	4	27
Sum	**37**	**5**	**42**	**126**	**12**	**138**
	Number of athletes 80+ years (Sweden)			Number of data points 80+ years (Sweden)		
Discipline	Men	Women	Total	Men	Women	Total
100 m	51	4	55	94	11	105
Long jump	31	5	36	83	10	93
Shot put	81	16	97	290	35	325
Discus throw	91	18	109	307	41	348
Javelin throw	68	9	77	248	15	263
Sum	**322**	**52**	**374**	**1,022**	**112**	**1,134**

### Decline Rates

**Figure 1:**
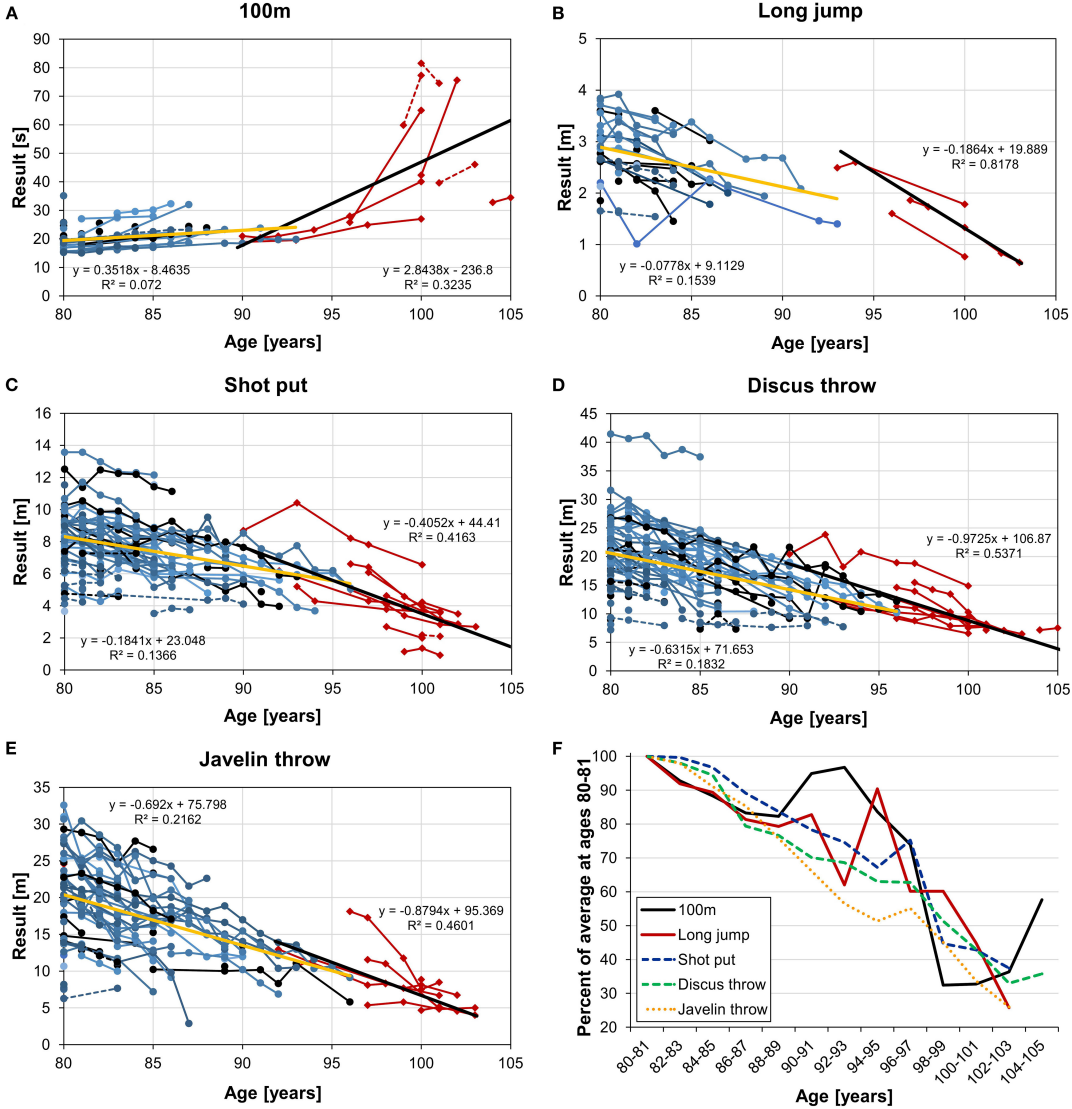
Longitudinal decline trajectories of individual athletes for 100-m sprint **(A)**, long jump **(B)**, shot put **(C)**, discus throw **(D)**, and javelin throw **(E)**. Dashed lines: women; solid lines: men. Blue/black lines and circles: data from the “Swedish Veteran Athletics” data base (Ganse et al., 2020b); red lines and rhombi: data of athletes with data at age 100 and/or older. Linear regression lines and functions are shown to allow comparison with the literature. Yellow line: regression line of data from “Swedish Veteran Athletics” (80+ years); black line: regression line of the new data from the “World Master Rankings” (100+ years). **(F)** Direct comparison of the performance declines normalized to ages 80–81 in the five disciplines, shown in pooled 2-year steps.

Figures 1A–E and Table 2 show that in all five disciplines the average decline rate was higher in the older compared to the younger athletes. The steepest increase in performance decline was found in the 100-m sprint (*t*-test, *p* < 0.05), where the decline rate was 8x as steep in the older compared to the younger group (Table 2). The least steep increase in performance decline was found in the javelin throw. The increases in decline were not significant in long jump and in the throws (*t*-test, Table 2). On average, the decline rate of centenarians was 2.53x as steep as compared to that between 80 and 96 years. Figure 1F shows a direct comparison of the disciplines.

**Table 2:** Comparison of decline rates between the 80+-years-old and centenarian group in seconds or meters per year and the ratio.

	**Linear regression, all data** **points pooled (R)**				**Decline rates all data** **points pooled (slopes)**			**Average individual** **decline rates (slopes)**			
**Discipline**	**R 80+**	**R Centenarians**	***p* 80+**	***p* Centenarians**	**80+ group**	**Centenarians**	**Ratio**	**80+ group**	**Centenarians**	**Ratio**	***P*-value (*t*-test)**
100 m	0.27	0.57	0.006	0.004	0.35	2.84	8.08	0.68 ± 0.86	5.59 ± 8.42	8.22	<0.05
Long jump	0.39	0.90	<0.001	<0.001	0.08	0.19	2.40	0.22 ± 0.45	0.18 ± 0.06	0.82	0.43
Shot put	0.38	0.65	<0.001	<0.001	0.18	0.51	2.74	0.23 ± 0.26	0.37 ± 0.21	1.61	0.06
Discus throw	0.43	0.73	<0.001	<0.001	0.63	0.97	1.54	0.79 ± 1.14	0.82 ± 0.70	1.04	0.47
Javelin throw	0.47	0.68	<0.001	<0.001	0.69	0.88	1.27	0.94 ± 0.67	0.92 ± 0.88	0.98	0.48
Average							**3.21**			**2.53**	

*The R and slope values were calculated on all data points pooled. “Average individual decline rates” were derived as the average of the individual regression slopes*.

### Sex Differences

Only 13.5% (5 out of 42 athletes) among the centenarians were women, which is in line with the 13.9% (52 out of 374 athletes) in the Swedish data set. Three women had results in the 100-m sprint, one in the shot put and one in the javelin throw. Due to the low number of women in the data-set, an analysis of sex differences in decline rates was not possible.

## Discussion

In the present study, we analyzed performance declines in five athletic disciplines of 42 master athletes who still performed at 100 years of age and older, and compared the data to the 80+-year-old athletes from a longitudinal data set we have published previously (Ganse et al., 2020b). The main finding of the study was that performance decline rates of centenarian athletes were on average 2.53-times as steep as the decline rate between 80 and 96 years of age. The fastest drop occurred in the 100-m sprint, while the javelin throw showed the least steep increase in the slope of decline. Of the centenarians, 13.5% were women, which is in line with the 13.9% in the younger collective.

### Performance Trends in Aging

It has been reported that the rate of performance decline was more than three times as fast in athletes older than 80 years compared to 30- to 69-year-old athletes (Ganse et al., 2020a,b). Accelerated decline rates after the age of 70 have also been shown in other disciplines and sports (Young and Starkes, 2005; Berthelot et al., 2012; Rubin et al., 2013; Dahl et al., 2020). The “plateau” in mortality rate in the oldest old (Barbi et al., 2018) may suggest a stabilization of the age-related rate of decrease in physical decline. Our data, however, do not show evidence for such a plateau in physical performance, but rather that the rate of decline is accelerated even further in 90+-year-old athletes beyond that seen in 80+-year-old athletes. Ongoing increases in decline rates were also previously reported from master world records (Baker and Tang, 2010; Akkari et al., 2015). At the same time, our data suggest that there is variation in the decline rate between individuals, that was also shown in the Swedish data set (Hoog Antink et al., 2021). Understanding the underlying factors for these differences could help to develop and improve interventions to slow down performance decline rates.

Differences in decline rates between track and field disciplines may be explained by specific skills required in each discipline, such as power, speed, agility, coordination or endurance, that may show different rates of age-related decline. For instance, the fastest performance decline was observed in the 100-m sprint that requires mainly speed and power (Dahl et al., 2019), while the javelin throw showed the least steep decline rate and depends besides power, on agility and coordination (Ganse and Degens, 2018). This needs to be studied in more detail, however, as previously we have observed that there was no significant difference in the rate of decline in aerobic and anaerobic power in master athletes (Bagley et al., 2019). It is clear that different physiological systems contribute to the success in different disciplines, where for instance power events are particularly dependent on muscle power, and endurance events are most limited by the cardiovascular system. It is therefore of interest to assess to what extent performance declines in different disciplines are attributable to proportional decrements in the muscle, respiratory (Degens et al., 2013), or cardiovascular function (Ganse and Degens, 2021).

### Centenarian Disciplines

Data from the oldest-old athletes were only available in 1 of the 10 individual running events, 1 of the 4 jumping events, but 3 of the 5 throwing events contested at the International Masters Games^1^. Interestingly, the event selection of athletes changes in the oldest old compared to earlier ages. Perhaps these disciplines were chosen, as the oldest-old athletes are physically unable to compete in other events. Such a limitation may already occur before the age of 100 years, as in another data set none of the 80–94-year-old athletes participated in hurdles or in other jumps (Ganse et al., 2020a). Possible factors that may contribute to the absence of oldest-old competitors in theses disciplines may be lack of sufficient muscle power and/or fear of injury.

### Sex Differences

Despite the higher life expectancy of women^2^, only 13.5% of the centenarian athletes in the present study were women. In comparison, 22% of the athletes in the largest longitudinal master athletics data set were women (Ganse et al., 2020b). Among the athletes with results at age 80 and older from the same data set, however, only 13.9% were women. The low proportion of oldest-old women may be related to a lower participation of women in sports in the past, where changes in society over the decades have stimulated a growing interest in sports and fitness in women, explaining a larger proportion of competing women at younger ages (O'Brien and Robertson, 2010).

### Strengths and Weaknesses

The main strength of the present study is that it is the first to present longitudinal data on changes in performance after the age of 95 of athletes still competing at the age of 100 or over. The main weakness is that a low amount of data from women led to the inability to test for sex differences. This is particularly interesting, as differences in decline rates between men and women have been shown in previous studies (Ganse et al., 2018; Gava et al., 2020). Larger data sets may show that the increases in decline between the younger and the older group are actually significant.

### Conclusions

We presented the first data set of performance decline rates after the age of 90 of athletes still competing at the age of 100 or over in five track and field disciplines. In this age group, the age-related performance decline was even faster than that previously reported in 80+-year-old athletes.

## Data Availability Statement

Publicly available datasets were analyzed in this study. This data can be found at: www.mastersrankings.com.

## Ethics Statement

Written informed consent was not obtained from the individual(s) for the publication of any potentially identifiable images or data included in this article.

## Author Contributions

BG contributed the idea and worked on data analysis and interpretation, figures, tables, drafting, and manuscript submission. HD contributed to the statistical analysis and data discussion. AB, CHA, MK, and TP helped with data interpretation. All authors have contributed to manuscript drafting and revision, read, and approved the submitted version of the manuscript.

## Funding

BG was funded by the German Research Foundation (DFG, grant number GA 2420/1-1).

## Conflict of Interest

The authors declare that the research was conducted in the absence of any commercial or financial relationships that could be construed as a potential conflict of interest.

## Publisher's Note

All claims expressed in this article are solely those of the authors and do not necessarily represent those of their affiliated organizations, or those of the publisher, the editors and the reviewers. Any product that may be evaluated in this article, or claim that may be made by its manufacturer, is not guaranteed or endorsed by the publisher.

## Footnotes

^1^ https://imga.ch/masters-sports/sports-and-disciplines/athletics/

^2^“Human Development Report”, United Nations Development Program. Available at http://hdr.undp.org/en/content/2019-human-development-index-ranking.
